# Global Mortality from Severe Infectious Diseases Among Adolescents Aged 10–19 Years, 1990–2023: Long-Term Trends and Cause Composition from the Global Burden of Disease 2023 Study

**DOI:** 10.3390/diseases14030094

**Published:** 2026-03-05

**Authors:** Young Joo Han

**Affiliations:** Division of Pediatric Critical Care Medicine, Department of Pediatrics, Yonsei University College of Medicine, Seoul 03722, Republic of Korea; yjhan@yuhs.ac

**Keywords:** adolescents, severe infectious diseases, mortality, Global Burden of Disease, COVID-19, socio-demographic index

## Abstract

Background: Severe infectious diseases remain a preventable cause of adolescent mortality worldwide, yet global evidence focused on adolescence as a distinct life-course stage—and its vulnerability to health system disruption—remains limited. We examined long-term mortality rate trends, cause composition, and COVID-19–related changes among adolescents compared with late childhood. Methods: We analyzed Global Burden of Disease 2023 mortality estimates from 1990 to 2023 for six acute severe infectious causes: lower respiratory infections, meningitis, encephalitis, diarrhoeal diseases, typhoid/paratyphoid fever, and COVID-19. Analyses focused on adolescents aged 10–19 years, with children aged 5–9 years as a comparator. Mortality rates (per 100,000 population) were the primary metric. Trends were quantified using estimated annual percentage change (EAPC), and pre-COVID, COVID peak, and post-COVID periods were compared across Socio-demographic Index (SDI) categories. Results: From 1990 to 2023, mortality rates declined globally across all age groups; however, reductions among adolescents were consistently slower than those among children aged 5–9 years (EAPC −2.27% vs. −3.55% per year). Diarrhoeal diseases and typhoid/paratyphoid fever exhibited the steepest long-term declines, whereas lower respiratory infections and meningitis demonstrated slower reductions and maintained a substantial share of adolescent mortality risk. During the COVID-19 peak, mortality rates modestly increased among adolescents, while children continued their gradual decline. Mortality rates remained highest in low-SDI settings. Conclusions: Despite substantial global progress, severe infectious diseases continue to impose significant and inequitable mortality risk among adolescents. The persistence of a concentrated cause profile and the amplification of mortality during system disruption underscore adolescence as a vulnerable life-course stage requiring sustained prevention and resilient acute care systems.

## 1. Introduction

Severe infectious diseases remain a major cause of preventable mortality in children and adolescents worldwide. Over the past three decades, substantial global progress in vaccination, sanitation, nutrition, and child health systems has led to marked declines in infection-related deaths, particularly among children under five years of age [1,2,3]. However, adolescents aged 10–19 years—often perceived as a generally healthy population and therefore receiving less targeted attention in global health planning—have experienced less pronounced improvements in mortality outcomes [4,5,6]. Despite lower absolute mortality compared with early childhood, adolescents continue to die from infections that are largely preventable and treatable, consistent with persistent gaps in timely recognition, access to acute care, and health system preparedness for this age group. These infections frequently manifest as acute respiratory failure, neurologic compromise, or septic shock requiring prompt recognition and urgent resuscitation. What remains less clear is how the cause-specific composition and the relative stability of severe infectious mortality among adolescents have been maintained or altered over time, particularly in response to major external shocks such as the COVID-19 pandemic. These uncertainties are particularly relevant in settings where socioeconomic and health systems constraints shape both exposure to infection and access to timely care.

Global analyses consistently show that infectious mortality among adolescents follows steep socioeconomic and geographic gradients [7,8,9]. Adolescents living in low Socio-demographic Index (SDI) settings face greater exposure to environmental and biological risks, delayed care-seeking, limited access to essential treatment, and systemic barriers that reduce survival from otherwise manageable infections. As young people transition from late childhood into adolescence—a developmental period characterized by declining preventive care utilization and increasing autonomy in health-seeking behavior—fragile health systems and structural inequities can create conditions in which severe infections progress rapidly to critical illness.

Regional experience illustrates the potential impact of health systems maturation. Several countries in East and Southeast Asia, for example, have achieved substantial reductions in infection-related mortality through expanded immunization programs, improved water and sanitation, and strengthened pediatric emergency and critical care capacity. These gains demonstrate the effectiveness of coordinated health system investments, while persistent within-country disparities underscore the fragility of progress. The COVID-19 pandemic further exposed vulnerabilities across all SDI levels: disruptions in routine immunization services, reduced access to acute care, and delays in care-seeking contributed to shifts in infection patterns and increased risks of severe outcomes among adolescents [10,11,12]. These vulnerabilities ultimately manifest through a limited set of severe infectious syndromes that account for most life-threatening presentations in adolescents.

Across settings, a small number of infectious syndromes consistently account for the majority of life-threatening presentations in older children and adolescents. Diarrhoeal diseases, lower respiratory infections, meningitis, encephalitis, and typhoid/paratyphoid fever remain prominent causes of severe illness, sepsis, and death in this age group. The emergence of COVID-19 introduced an additional high-impact pathogen with both direct and indirect effects on adolescent morbidity and mortality. These conditions share common biological pathways—including hypoxia, circulatory collapse, and systemic inflammation—that frequently necessitate timely diagnosis, antimicrobial therapy, and access to higher-level emergency or critical care services. Nevertheless, comprehensive evidence describing long-term trends, cause-specific composition, and socioeconomic inequalities in severe infection mortality among adolescents remains limited.

Most global analyses of infectious mortality have focused primarily on younger children, maternal and neonatal populations, or all-age aggregates. Consequently, several key questions remain unanswered: How has severe infection mortality evolved among adolescents over the past three decades? How do mortality patterns differ by age subgroup and level of socioeconomic development? To what extent did the COVID-19 pandemic disrupt pre-existing trends? And which infections contribute most substantially to adolescent critical illness over time?

To address these gaps, we analyzed global mortality from six major severe infectious diseases—diarrhoeal diseases, lower respiratory infections, meningitis, encephalitis, typhoid/paratyphoid fever, and COVID-19 [13,14,15]—among adolescents aged 10–19 years from 1990 to 2023, using data from the Global Burden of Disease (GBD) Study. We examined long-term trends across age subgroups, compared patterns by SDI level, and evaluated changes across pre-pandemic, pandemic, and post-pandemic periods. By characterizing epidemiologic trajectories and socioeconomic disparities in severe infection mortality, this study aims to inform strategies to reduce preventable infectious deaths and to strengthen adolescent-responsive emergency and critical care systems.

## 2. Methods

This study used publicly available cause-specific mortality estimates from the GBD study, a standardized framework designed to enable comparable mortality estimation across locations and over time [6,16,17]. All data were aggregated and de-identified; therefore, institutional review board approval was not required. Both absolute death counts and mortality rates (per 100,000 population) were analyzed, with mortality rates serving as the primary outcome measure for comparative analyses and absolute death counts providing contextual information on population burden.

Age categories were defined according to the standard GBD age framework. Adolescents were analyzed as ages 10–19 years, with subgroup analyses for early adolescence (10–14 years) and late adolescence (15–19 years). These categories are consistent with the World Health Organization definition of adolescence as ages 10–19 years and reflect established public health classifications rather than strictly biologic pubertal thresholds [18,19,20].

We examined six infectious causes relevant to life-threatening illness in older children and adolescents: diarrhoeal diseases, lower respiratory infections, meningitis, encephalitis, typhoid/paratyphoid fever, and COVID-19. In the GBD cause hierarchy, these conditions are treated as underlying causes of death based on ICD-coded cause-of-death data and harmonized attribution methods, enabling consistent comparisons across settings and years [1,16,17].

These causes were selected a priori to represent acute, high-severity infectious syndromes consistently associated with rapid clinical deterioration and critical care utilization in pediatric and adolescent populations. Lower respiratory infections, meningitis, encephalitis, and severe diarrhoeal disease are consistently identified as leading causes of infection-related critical illness and sepsis in hospital-based and intensive care cohorts worldwide, particularly in resource-limited settings [21]. In contrast, chronic infections such as tuberculosis or human immunodeficiency virus (HIV) were not included because their mortality pathways and healthcare trajectories differ substantially from acute syndromic infections and would not directly reflect patterns of severe, rapidly progressive infectious mortality.

Sepsis was not analyzed as a standalone cause because, within the GBD framework, sepsis is modeled as a clinical syndrome arising from multiple underlying etiologies rather than as an underlying cause of death; accordingly, sepsis-related deaths are attributed to the precipitating infectious causes [22,23].

To evaluate pandemic-related changes, we defined three periods: pre-COVID (2015–2019), COVID peak (2020–2021), and post-COVID (2022–2023), consistent with the timing of major global transmission waves and health system disruption [10,12].

Analyses were conducted globally and by SDI level (low, low-middle, middle, and high-middle-SDI). SDI is a composite measure incorporating income per capita, educational attainment, and fertility, and is used within the GBD framework to summarize socioeconomic development [7].

Long-term temporal trends were quantified using the estimated annual percentage change (EAPC). Annual mortality rates were modeled using log-linear regression with calendar year as the independent variable, and EAPC was calculated as (e^β − 1) × 100 [24].

All analyses were performed using R version 4.3.0 (R Foundation for Statistical Computing, Vienna, Austria). Figures were finalized using GraphPad Prism version 10 (GraphPad Software, San Diego, CA, USA).

## 3. Results

### 3.1. Global Trends in Mortality from Severe Infectious Diseases

**Figure 1:**
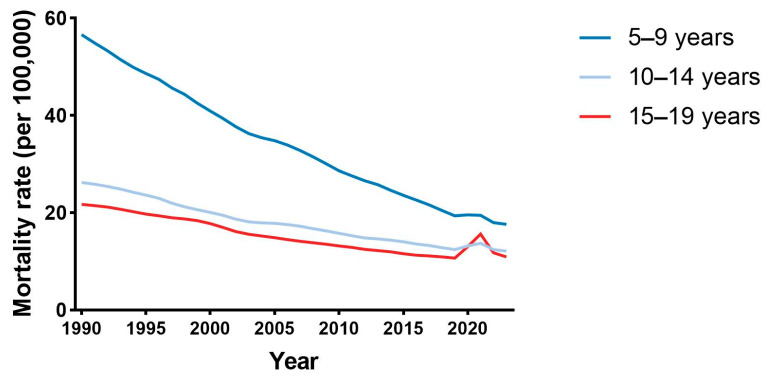
Global mortality rates from six severe infectious diseases by age group, 1990–2023. Lines show annual mortality rates (per 100,000 population) for the combined six severe infectious causes—lower respiratory infections, meningitis, encephalitis, diarrhoeal diseases, typhoid/paratyphoid fever, and COVID-19—across four age groups (10–14, 15–19, 10–19, and 5–9 years).

From 1990 to 2023, global mortality rates (per 100,000 population) from the six severe infectious diseases declined substantially across all age groups (Figure 1). Although reductions were observed among adolescents aged 10–19 years, the pace of decline was consistently slower than that seen in children aged 5–9 years. Mortality rates remained higher among older adolescents (15–19 years) than younger adolescents (10–14 years) throughout most of the study period.

During the COVID-19 period, modest age-differential deviations from long-term trends were observed. Mortality rates among older adolescents (15–19 years) rose slightly between 2019 and 2021, followed by partial attenuation in 2023. In contrast, rates among younger adolescents (10–14 years) remained broadly stable, while children aged 5–9 years continued their pre-pandemic downward trajectory with only minor fluctuation.

Corresponding absolute death counts followed patterns broadly consistent with these rate-based trends and are provided in Supplementary Table S1 for contextual reference.

### 3.2. Cause-Specific Mortality Patterns Among Adolescents

**Figure 2:**
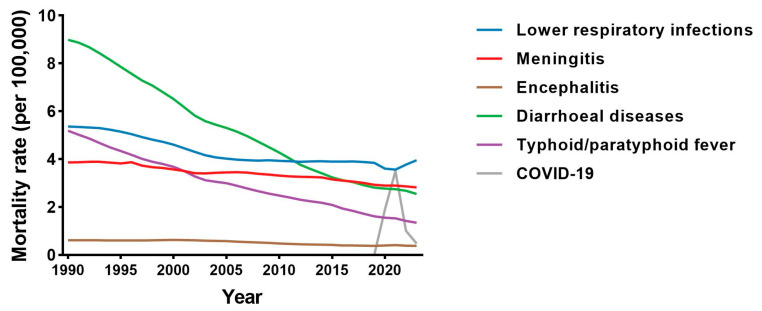
Cause-specific mortality rates from six severe infectious diseases among adolescents aged 10–19 years, 1990–2023 (global). Lines represent annual mortality rates (per 100,000 population) for lower respiratory infections, meningitis, encephalitis, diarrhoeal diseases, typhoid/paratyphoid fever, and COVID-19.

Cause-specific mortality rates among adolescents aged 10–19 years demonstrated substantial heterogeneity over time (Figure 2). Diarrhoeal diseases and typhoid/paratyphoid fever showed the steepest long-term rate reductions between 1990 and 2023, whereas lower respiratory infections and meningitis declined more modestly and maintained a relatively stable contribution to overall mortality risk. Encephalitis demonstrated a greater proportional decline than lower respiratory infections and meningitis, although absolute rate differences were smaller given its lower baseline mortality. COVID-19 was associated with a sharp but time-limited increase in mortality rates during 2020–2021, followed by partial attenuation in 2022–2023.

Absolute numbers of cause-specific deaths (Table 1) followed similar directional trends, with large proportional reductions in diarrhoeal diseases (−64.5%) and typhoid/paratyphoid fever (−67.5%), and comparatively smaller reductions for lower respiratory infections (−7.4%) and meningitis (−8.4%). COVID-19 contributed substantial mortality during 2020–2021, with lower but persistent deaths in 2023.

### 3.3. Socio-Demographic Disparities in Adolescent Mortality

**Figure 3:**
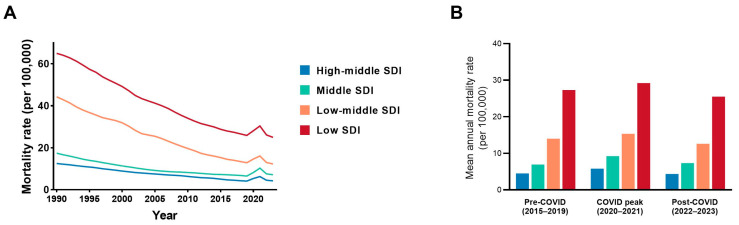
Socio-demographic disparities in mortality rates from six severe infectious diseases among adolescents aged 10–19 years, 1990–2023 (global). (A) Annual mortality rates (per 100,000 population) stratified by Socio-demographic Index (SDI) level (high-middle, middle, low-middle, and low-SDI). (B) Mean annual mortality rates (per 100,000 population) by SDI level across three predefined periods: pre-COVID (2015–2019), COVID peak (2020–2021), and post-COVID (2022–2023). Colors represent SDI categories: High-middle SDI (blue), Middle SDI (green), Low-middle SDI (orange), and Low SDI (red).

Mortality rates declined across all SDI categories from 1990 to 2019; notably, the steepest relative declines were observed in low- and low-middle-SDI settings. Despite these larger relative reductions, mortality rates in low-SDI regions remained the highest throughout the study period (Figure 3A).

During the COVID-19 period, mortality rates showed modest fluctuations across SDI categories, without substantial alteration in the relative ranking between groups (Figure 3B). In 2023, adolescents in low-SDI settings continued to experience substantially higher mortality rates across most infectious causes (Supplementary Table S2), and absolute mortality counts reflected a similar concentration of burden (Supplementary Table S3).

### 3.4. Changes Before, During, and After the COVID-19 Pandemic

For all COVID-period analyses, the pre-COVID period was defined as 2015–2019, the COVID peak period as 2020–2021, and the post-COVID period as 2022–2023.

**Figure 4:**
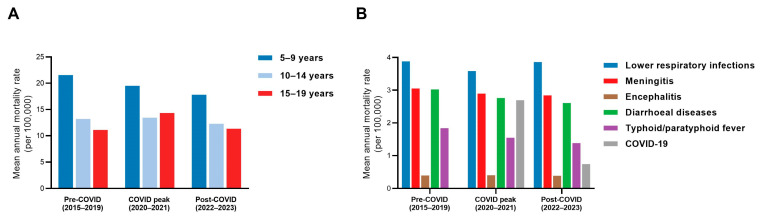
Changes in mortality rates from six severe infectious diseases before, during, and after the COVID-19 pandemic (global). (**A**) Mean annual mortality rates (per 100,000 population) by age group (10–14, 15–19, 10–19, and 5–9 years) across three predefined periods: pre-COVID (2015–2019), COVID peak (2020–2021), and post-COVID (2022–2023). (**B**) Mean annual mortality rates by cause among adolescents aged 10–19 years across the same periods, including lower respiratory infections, meningitis, encephalitis, diarrhoeal diseases, typhoid/paratyphoid fever, and COVID-19. Bars represent mean annual mortality rates within each period.

Mortality rates showed a modest increase during the COVID peak period among older adolescents (15–19 years), whereas younger adolescents (10–14 years) experienced minimal change (Figure 4A). In contrast, mortality rates among children aged 5–9 years continued their gradual decline, with only minor pandemic-period fluctuation.

Cause-specific rate patterns differed across infections. Lower respiratory infection mortality rates decreased during the COVID peak period, followed by a rebound thereafter. Encephalitis showed a modest increase relative to pre-pandemic levels, while meningitis remained comparatively stable. Diarrhoeal diseases and typhoid/paratyphoid fever continued to decline. COVID-19 contributed substantially to mortality rates during the peak period, with lower but persistent levels thereafter (Figure 4B). Absolute mean annual deaths across periods are provided in Supplementary Tables S4 and S5 to contextualize changes in overall population burden.

### 3.5. Long-Term Temporal Trends

Long-term trends in mortality rates were quantified using EAPC (Supplementary Figure S1A; Supplementary Table S6). Children aged 5–9 years experienced the steepest decline (EAPC −3.55% per year), followed by adolescents aged 10–14 years (−2.38% per year) and 15–19 years (−2.13% per year). The overall EAPC for adolescents aged 10–19 years was −2.27% per year.

Cause-specific EAPC estimates further confirmed heterogeneity in rate trajectories (Supplementary Figure S1B; Supplementary Table S7). Diarrhoeal diseases and typhoid/paratyphoid fever exhibited the most rapid long-term declines, whereas lower respiratory infections, meningitis, and encephalitis demonstrated slower but statistically significant decreases. EAPC was not calculated for COVID-19 because mortality occurred only after 2020 and did not represent a sustained long-term temporal trajectory suitable for log-linear modeling.

Temporal patterns in cause-specific mortality rates (Supplementary Figure S2) illustrate a progressive attenuation beginning in the early 1990s, interrupted by a transient increase during the COVID-19 period and followed by a return toward pre-pandemic levels.

Sex-stratified analyses demonstrated declining mortality rates in both males and females, with slightly steeper long-term reductions observed among males (Supplementary Table S8).

## 4. Discussion

In this global analysis spanning more than three decades, mortality rates from six severe infectious diseases among adolescents aged 10–19 years declined between 1990 and 2023; however, the pace and structure of these declines varied by age group, cause, and socio-demographic context. Although overall mortality risk decreased, reductions among adolescents were consistently slower than those observed in children aged 5–9 years. Moreover, a limited set of causes—most notably lower respiratory infections and meningitis—continued to account for a substantial proportion of adolescent deaths across the study period. The COVID-19 pandemic functioned as a major external shock that disrupted long-standing trends, with disproportionate increases in mortality risk among older adolescents and in low-SDI settings. Collectively, these findings suggest that adolescent severe infectious mortality has improved over time yet remains concentrated within a limited cause profile that is sensitive to system-level disruption [9,17,19]. By examining adolescence as a distinct life-course stage rather than aggregating across broader pediatric age groups, this study underscores patterns of slower risk reduction, concentrated cause stability, and heightened vulnerability to system disruption that may be obscured in all-age or under-five analyses.

A central observation is the slower decline in mortality rates among adolescents compared with late childhood, particularly among those aged 15–19 years. Rather than implying a single explanatory mechanism, this divergence is consistent with adolescence representing a transitional life-course stage in which gains achieved through early-childhood prevention and health system investments may not be fully sustained. Prior adolescent health frameworks emphasize shifts in healthcare engagement and increasing autonomy in care-seeking during this period, which may plausibly influence patterns of presentation and treatment for acute illness. While such mechanisms cannot be directly inferred from ecological GBD estimates, the persistence of differential mortality risk suggests that adolescence warrants distinct consideration within infectious disease policy and health system planning [19,20].

The six infectious causes were selected a priori to represent acute, life-threatening syndromes consistently identified in pediatric and adolescent critical care as drivers of rapid clinical deterioration and infection-related mortality, rather than chronic infections with distinct epidemiologic trajectories and care pathways. For transparency, the full list of GBD Level 3 communicable diseases and the corresponding rationale for exclusion from this acute syndromic framework are summarized in Supplementary Table S9.

Within this framework, it is important to recognize the microbiologic diversity underlying these syndromes. These syndromic categories encompass well-characterized bacterial and viral pathogens widely recognized in pediatric and adolescent populations, including agents responsible for lower respiratory infections (e.g., *Streptococcus pneumoniae*, influenza viruses), meningitis (e.g., *Neisseria meningitidis*, *Streptococcus pneumoniae*), enteric pathogens causing diarrhoeal diseases, *Salmonella Typhi* in typhoid fever, and SARS-CoV-2 in COVID-19. Despite microbiologic heterogeneity, these conditions converge clinically in their potential for rapid physiologic deterioration—manifesting as respiratory failure, neurologic compromise, circulatory instability, or severe dehydration—and therefore require timely recognition and effective acute management.

Cause-specific analyses revealed two distinct long-term trajectories. Mortality rates from diarrhoeal diseases and typhoid/paratyphoid fever declined markedly and consistently between 1990 and 2023, representing the steepest reductions among the six infectious causes examined. In contrast, lower respiratory infections and meningitis declined only modestly over the same period and continued to account for a stable and prominent share of adolescent infectious mortality.

Within this acute syndromic framework, the comparatively limited reduction in respiratory and neurologic infections suggests persistent vulnerability despite overall global progress in infectious disease control. These contrasting patterns may reflect differential responsiveness to public health interventions: enteric infections have likely benefited from sustained improvements in sanitation, vaccination, and antimicrobial access, whereas lower respiratory infections and meningitis frequently depend on timely recognition, effective referral systems, and reliable access to acute care. Although the present ecological analysis cannot directly attribute mortality trends to specific health system mechanisms, the time-sensitive nature of these syndromes provides a clinically coherent context for their continued contribution to adolescent mortality risk [13,25,26,27,28].

Pronounced socio-demographic gradients were observed throughout the study period. Adolescents in low-SDI settings experienced consistently higher mortality rates than those in higher-SDI regions. Although lower-SDI settings exhibited the steepest relative declines in mortality over time, rates remained persistently higher, reflecting narrowing but enduring disparities. These differences should be interpreted descriptively rather than causally; however, they are consistent with broader global health evidence indicating that survival gaps across socio-demographic contexts often correspond to differences in exposure risk, healthcare access, and treatment effectiveness. Periods of system stress may influence these patterns [9,19,20]; in the present analysis, pandemic-period fluctuations were modest and did not substantially alter the overall SDI gradient.

The COVID-19 period was associated with a modest and time-limited deviation from long-term mortality trends among adolescents. Changes were small and varied by age group, while children largely maintained their pre-pandemic trajectory. These findings suggest that adolescent infectious mortality patterns may exhibit some sensitivity to external system disruptions, although the magnitude of change was limited [10,12,29].

These findings have practical implications for adolescent health policy and service design. The persistence of mortality risk in lower respiratory infections and meningitis suggests that strengthening core components of acute care—such as reliable oxygen delivery systems, timely administration of parenteral antimicrobials, and effective referral pathways for rapidly deteriorating respiratory or neurologic illness—may directly address the syndromes that continue to dominate adolescent infectious mortality.

In parallel, sustaining adolescent immunization coverage and safeguarding routine preventive services during periods of system disruption may help mitigate amplification of mortality risk under stress conditions. Explicitly incorporating adolescents into health system preparedness and emergency planning frameworks may therefore enhance both resilience and equity in infection-related survival.

Taken together, these findings suggest that adolescent infectious mortality has declined more slowly than in late childhood, remains concentrated in a small set of high-impact syndromes, and is disproportionately disrupted during periods of system stress—particularly in low-SDI settings [9,19,20,25,26,27,28].

## 5. Strengths and Limitations

This study leveraged standardized and internally consistent GBD 2023 estimates spanning more than three decades, enabling comparable assessment of mortality rates across time, age groups, causes, and SDI strata. By explicitly focusing on adolescence, the analysis addresses a persistent gap in global infectious mortality reporting, which often prioritizes under-five populations or aggregates across all ages [17,19].

Several limitations warrant consideration. First, GBD estimates are model-based and depend on the availability and quality of underlying data. In settings with limited vital registration systems, greater reliance on statistical redistribution and model assumptions may introduce uncertainty and potential misclassification between clinically overlapping infectious causes. Such limitations may differentially affect adolescent cause-of-death estimates compared with younger children, particularly in regions with sparse data, and should be considered when interpreting cross-age or cross-SDI comparisons.

Second, the ecological design precludes individual-level inference, and associations between mortality rates and socio-demographic context should be interpreted as descriptive patterns rather than causal estimates of health system performance. Third, the analysis is restricted to mortality and does not capture non-fatal severe morbidity or pathway-level determinants that may influence outcomes. Finally, predefined pandemic periods may not fully capture regional heterogeneity in COVID-19 timing and intensity. Despite these limitations, the long-term and globally comparable nature of the data enables identification of age-specific patterns and structural vulnerabilities relevant to adolescent health policy and system resilience [10,12,17].

## 6. Conclusions

Severe infectious diseases remain an important and inequitable cause of mortality risk among adolescents worldwide. The persistence of a concentrated cause composition and the amplification of mortality risk during periods of system disruption—particularly in low-SDI settings—underscore the need to integrate sustained infection prevention with strengthened, adolescent-responsive acute care systems [9,19,20,25,26,27,28].

## Figures and Tables

**Table 1:** Cause-specific mortality from six severe infectious diseases among adolescents aged 10–19 years in selected years, 1990–2023.

Cause of Death	1990	2010	2019	2021	2023	% Change (1990→2023)
Lower respiratory infections	56,711	47,626	48,736	46,245	52,506	−7.41%
Meningitis	40,872	40,161	37,169	37,534	37,453	−8.37%
Encephalitis	6496	5862	4829	5325	5048	−22.30%
Diarrhoeal diseases	95,089	51,862	35,673	35,649	33,730	−64.53%
Typhoid/paratyphoid fever	54,819	30,077	20,457	19,847	17,821	−67.49%
COVID-19	0	0	0	45,445	6381	—

Deaths are presented for six major life-threatening infectious causes—lower respiratory infections, meningitis, encephalitis, diarrhoeal diseases, typhoid/paratyphoid fever, and COVID-19—at representative years capturing long-term trends (1990 and 2010), the pre-COVID baseline (2019), the pandemic peak (2021), and the most recent post-pandemic year (2023). Percentage change represents the relative change in cause-specific mortality between 1990 and 2023. Percentage change was not calculated for COVID-19 because deaths occurred only after 2020.

## Data Availability

The data are publicly available through the Global Burden of Disease 2023 Results Tool (IHME), and no new data were generated in this study.

## Supplementary Materials

The following supporting information can be downloaded at: https://www.mdpi.com/article/10.3390/diseases14030094/s1, Figure S1: Estimated annual percentage change (EAPC) in mortality rates from severe infectious diseases, 1990–2023 (Global); Figure S2: Temporal intensity of mortality rates (per 100,000 population) from six severe infectious diseases among adolescents aged 10–19 years, 1990–2023 (Global); Table S1: Global absolute deaths from six severe infectious diseases by age group at selected years, 1990–2023; Table S2: Mortality rates from six severe infectious diseases among adolescents aged 10–19 years by Socio-demographic Index (SDI) level, 2023 (per 100,000 population); Table S3: Mortality counts from six severe infectious diseases among adolescents aged 10–19 years by Socio-demographic Index (SDI) level, 2023; Table S4: Mean annual deaths from six severe infectious diseases by age group across pre-COVID, COVID peak, and post-COVID periods (global): Table S5: Mean annual deaths from six severe infectious diseases among adolescents aged 10–19 years across pre-COVID, COVID peak, and post-COVID periods (global); Table S6: Estimated annual percentage change (EAPC) in mortality rates from six severe infectious diseases by age group, 1990–2023 (global); Table S7: Cause-specific estimated annual percentage change (EAPC) in mortality rates from six severe infectious diseases among adolescents aged 10–19 years, 1990–2023 (global); Table S8: Sex-stratified mortality rates and long-term temporal trends among adolescents aged 10–19 years, 1990–2023 (global); Table S9: GBD 2023 Level 3 communicable diseases and rationale for exclusion from the present acute life-threatening infectious framework.
