# Supplementary material for: Global Mortality from Severe Infectious Diseases Among Adolescents Aged 10–19 Years, 1990–2023: Long-Term Trends and Cause Composition from the Global Burden of Disease 2023 Study
Source: Diseases. 2026 Mar 5;14(3):94. doi: 10.3390/diseases14030094 (PMC13024756; doi:10.3390/diseases14030094)
Supplement: Supplementary file 1 [file diseases-14-00094-s001.zip › diseases-4128176-supplementary.pdf]

Supplementary Materials

Contents

Supplementary Methods

1. COVID-19 period definitions

2. Socio-demographic Index (SDI)

3. Trend estimation

Supplementary Figures

Figure S1. Estimated annual percentage change (EAPC) in mortality rates from severe infectious diseases, 1990–2023 (Global).

Figure S2. Temporal intensity of mortality rates (per 100,000 population) from six severe infectious diseases among adolescents aged 10–19 years, 1990–2023 (Global).

Supplementary Tables

Table S1. Global absolute deaths from six severe infectious diseases by age group at selected years, 1990–2023.

Table S2. Mortality rates from six severe infectious diseases among adolescents aged 10–19 years by Socio-demographic Index (SDI) level, 2023 (per 100,000 population).

Table S3. Mortality counts from six severe infectious diseases among adolescents aged 10–19

years by Socio-demographic Index (SDI) level, 2023.

Table S4. Mean annual deaths from six severe infectious diseases by age group across pre-COVID, COVID peak, and post-COVID periods (global).

Table S5. Mean annual deaths from six severe infectious diseases among adolescents aged 10–19 years across pre-COVID, COVID peak, and post-COVID periods (global).

Table S6. Estimated annual percentage change (EAPC) in mortality rates from six severe infectious diseases by age group, 1990–2023 (global).

Table S7. Cause-specific estimated annual percentage change (EAPC) in mortality rates from six severe infectious diseases among adolescents aged 10–19 years, 1990–2023 (global).

Table S8. Sex-stratified mortality rates and long-term temporal trends among adolescents aged 10–19 years, 1990–2023 (global).

Table S9. GBD 2023 Level 3 communicable diseases and rationale for exclusion from the present acute life-threatening infectious framework

Supplementary Methods

COVID-19 period definitions

To evaluate pandemic-related changes in mortality, three predefined periods were used. The pre-COVID period was defined as 2015–2019, representing a stable baseline prior to the COVID-19 pandemic. The COVID peak period was defined as 2020–2021, corresponding to major global transmission waves and widespread health system disruption. The post-COVID period was defined as 2022–2023, reflecting partial recovery of health services and stabilization of mortality patterns. Mean annual mortality rates were calculated within each period.

Socio-demographic Index (SDI)

The Socio-demographic Index (SDI) is a composite measure developed within the Global Burden of Disease framework that incorporates lag-distributed income per capita, average educational attainment among adults aged ≥15 years, and total fertility rate under age 25. Countries and territories are typically classified into five SDI categories (low, low-middle, middle, high-middle, and high SDI) within the GBD framework. However, the GBD 2023 dataset downloaded at the time of analysis provided SDI-stratified estimates aggregated into four categories (low, low-middle, middle, and high-middle SDI). Accordingly, results are presented using these four SDI strata.

Trend estimation

Long-term temporal trends were quantified using the estimated annual percentage change (EAPC). Annual mortality rates were modeled using log-linear regression, with calendar year as the independent variable. EAPC values were calculated as $(e^{\beta} - 1) \times 100$, where β represents the regression coefficient. Corresponding p-values were derived from the regression model.

Supplementary Figures

Figure S1. Estimated annual percentage change (EAPC) in mortality rates from severe infectious diseases, 1990–2023 (Global).

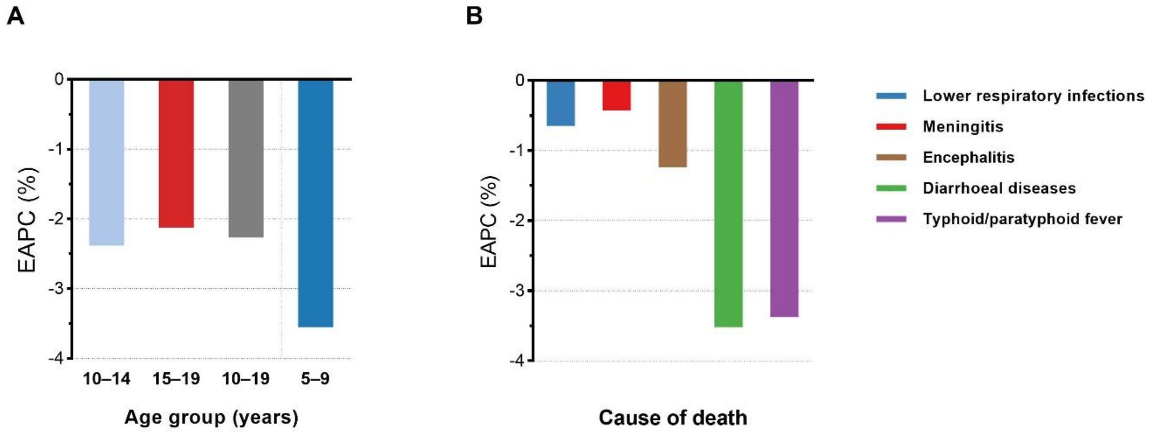

EAPC values were derived using log-linear regression models fitted to annual mortality rates (per 100,000 population) from 1990 to 2023. Negative values indicate declining mortality rates over time.

(A) EAPC in mortality rates from the combined total of six severe infectious diseases—lower respiratory infections, meningitis, encephalitis, diarrhoeal diseases, typhoid/paratyphoid fever, and COVID-19—across four age groups (10–14, 15–19, 10–19, and 5–9 years).

(B) Cause-specific EAPC in mortality rates from severe infectious diseases among adolescents aged 10–19 years. EAPC was not calculated for COVID-19 because mortality occurred only after 2020 and did not represent a sustained long-term temporal trajectory suitable for log-linear modeling.

Figure S2. Temporal intensity of mortality rates (per 100,000 population) from six severe infectious diseases among adolescents aged 10–19 years, 1990–2023 (Global).

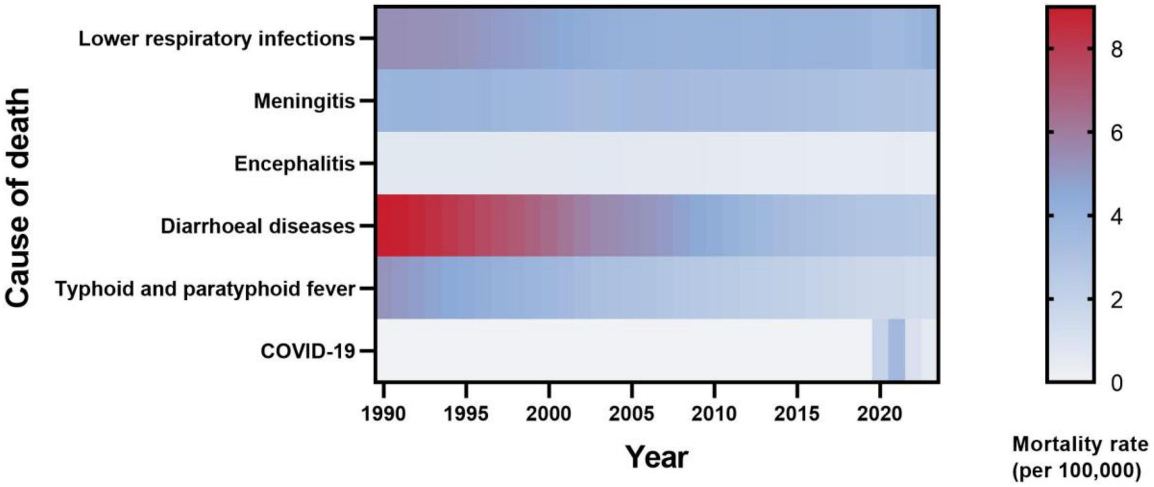

This heatmap depicts annual mortality rates (per 100,000 population) from six severe infectious diseases—lower respiratory infections, meningitis, encephalitis, diarrhoeal diseases, typhoid/paratyphoid fever, and COVID-19—among adolescents aged 10–19 years from 1990 to 2023. Rows represent infectious causes and columns represent calendar years. Color intensity corresponds to mortality rate magnitude, illustrating heterogeneous long-term trajectories across causes and the pandemic-associated increase during 2020–2021.

Supplementary Tables

Table S1. Global absolute deaths from six severe infectious diseases by age group at selected years, 1990–2023.

Year	5–9 y	10–14 y	15–19 y	10–19 y
1990	330,125	140,767	113,221	253,988
2000	246,440	125,415	101,321	226,736
2010	176,366	95,308	80,280	175,588
2019	132,056	81,405	65,460	146,864
2021	135,388	92,142	97,904	190,046
2023	123,455	82,672	70,267	152,940

Note:

Mortality represents the combined number of deaths from six severe infectious diseases—lower respiratory infections, meningitis, encephalitis, diarrhoeal diseases, typhoid/paratyphoid fever, and COVID-19—allowing comparison of long-term mortality trends between adolescence and late childhood. Deaths are shown for four age groups (10–14, 15–19, 10–19, and 5–9 years) at selected time points spanning the pre-COVID period (2019), the COVID-19 peak (2021), and the most recent post-pandemic year (2023), as well as earlier reference years (1990, 2000, and 2010).

Table S2. Mortality rates from six severe infectious diseases among adolescents aged 10–19 years by Socio-demographic Index (SDI) level, 2023 (per 100,000 population).

**SDI level**	**Lower respiratory infections**	**Meningitis**	**Encephalitis**	**Diarrhoeal diseases**	**Typhoid/ paratyphoid fever**	**COVID-19**
Global	3.96	2.82	0.38	2.54	1.34	0.48
High-middle SDI	1.66	0.47	0.32	0.78	0.60	0.37
Middle SDI	2.96	1.18	0.49	0.82	0.74	0.98
Low-middle SDI	3.33	2.59	0.64	2.86	2.58	0.28
Low SDI	8.47	7.12	0.42	6.03	2.29	0.59

Note:

Mortality rates are expressed per 100,000 population for six severe infectious diseases among adolescents aged 10–19 years in 2023. Estimates are shown for the global total and across four SDI categories (high-middle, middle, low-middle, and low SDI).

Table S3. Mortality counts from six severe infectious diseases among adolescents aged 10–19 years by Socio-demographic Index (SDI) level, 2023.

**SDI level**	**Lower respiratory infections**	**Meningitis**	**Encephalitis**	**Diarrhoeal diseases**	**Typhoid/ paratyphoid fever**	**COVID-19**
Global	52,506	37,453	5,048	33,730	17,821	6,381
High-middle SDI	4,071	1,152	776	1,916	1,472	893
Middle SDI	4,871	1,943	801	1,350	1,217	1,609
Low-middle SDI	7,738	6,015	1,484	6,647	5,996	650
Low SDI	33,166	27,893	1,637	23,611	8,957	2,292

Note:

This table presents the number of deaths from six severe infectious diseases—lower respiratory infections, meningitis, encephalitis, diarrhoeal diseases, typhoid/paratyphoid fever, and COVID-19—among adolescents aged 10–19 years in 2023. Estimates are shown for the global total and across four SDI categories (high-middle, middle, low-middle, and low SDI).

Table S4. Mean annual deaths from six severe infectious diseases by age group across pre-COVID, COVID peak, and post-COVID periods (global).

**Age group**	**Pre (2015–2019)**	**COVID peak (2020–2021)**	**Post (2022–2023)**	**% change Pre→COVID**	**% change Pre→Post**
10–14 y	14,031	14,968	13,900	+6.7%	−0.9%
15–19 y	11,224	14,878	12,090	+32.6%	+7.7%
10–19 y combined	25,255	29,847	25,990	+18.2%	+2.9%
5–9 y	24,061	22,496	20,785	−6.5%	−13.6%

Note:

Values represent the mean annual number of deaths from six severe infectious diseases—lower respiratory infections, meningitis, encephalitis, diarrhoeal diseases, typhoid and paratyphoid fever, and COVID-19—calculated for three predefined periods: pre-COVID (2015–2019), COVID peak (2020–2021), and post-COVID (2022–2023). Estimates are shown for four age groups (10–14, 15–19, 10–19, and 5–9 years). Percentage changes are calculated relative to the pre-COVID period.

Table S5. Mean annual deaths from six severe infectious diseases among adolescents aged 10–19 years across pre-COVID, COVID peak, and post-COVID periods (global).

**Cause of death**	**Pre (2015–2019)**	**COVID peak (2020–2021)**	**Post (2022–2023)**	**% change Pre→COVID**	**% change Pre→Post**
Lower respiratory infections	48,275	46,230	50,970	−4.23%	+5.58%
Meningitis	37,934	37,329	37,509	−1.59%	−1.12%
Encephalitis	4,911	5,193	5,079	+5.74%	+3.41%
Diarrhoeal diseases	37,570	35,604	34,413	−5.23%	−8.40%
Typhoid/paratyphoid fever	22,838	19,903	18,206	−12.9%	−20.3%
COVID-19	0	34,820	9,764	—	—

Note:

Values represent the mean annual number of deaths among adolescents aged 10–19 years, summarized by cause for three predefined periods: pre-COVID (2015–2019), COVID peak (2020–2021), and post-COVID (2022–2023). Percentage changes are calculated relative to the pre-COVID period. Percentage change was not calculated for COVID-19 because deaths occurred only after 2020.

Table S6. Estimated annual percentage change (EAPC) in mortality rates from six severe infectious diseases by age group, 1990–2023 (global).

**Age group**	**EAPC, % per year (95% CI)**	***p*-value**
10–14 years	−2.382 (−2.488 to −2.275)	5.00×10^{-30}
15–19 years	−2.126 (−2.410 to −1.842)	1.26×10^{-15}
10–19 years	−2.267 (−2.454 to −2.079)	1.04×10^{-21}
5–9 years	−3.553 (−3.622 to −3.484)	2.25×10^{-41}

Note:

Estimated annual percentage change (EAPC) values were derived from log-linear regression models fitted to annual mortality rates (per 100,000 population) from 1990 to 2023. Negative EAPC values indicate decreasing mortality trends over time. Estimates are presented for four age groups (10–14, 15–19, 10–19, and 5–9 years).

Table S7. Cause-specific estimated annual percentage change (EAPC) in mortality rates from six severe infectious diseases among adolescents aged 10–19 years, 1990–2023 (global).

**Cause of death**	**EAPC, % per year (95% CI)**	***p*-value**
Lower respiratory infections	−1.190 (−1.359 to −1.022)	5.66×10^{-15}
Meningitis	−0.972 (−1.033 to −0.910)	2.39×10^{-25}
Encephalitis	−1.775 (−1.996 to −1.554)	1.57×10^{-16}
Diarrhoeal diseases	−4.046 (−4.149 to −3.942)	1.44×10^{-37}
Typhoid/paratyphoid fever	−3.903 (−4.004 to −3.802)	1.90×10^{-37}
COVID-19	—	—

Note:

Cause-specific EAPC values were derived from log-linear regression models fitted to annual mortality rates (per 100,000 population) from 1990 to 2023. Negative EAPC values indicate decreasing mortality trends over time.

EAPC was not calculated for COVID-19 because mortality occurred only after 2020 and did not represent a long-term temporal trend suitable for log-linear modeling.

Table S8. Sex-stratified mortality rates and long-term temporal trends among adolescents aged 10–19 years, 1990–2023 (global).

**Sex**	**Mortality rate 1990**	**Mortality rate 2023**	**Absolute change**	**EAPC, % per year (95% CI)**	**p-value**
Male	23.44	10.87	−12.58	−2.41 (−2.60 to −2.23)	<0.001
Female	24.61	12.22	−12.39	−2.12 (−2.33 to −1.91)	<0.001

Note:

Mortality rates are expressed per 100,000 population. EAPC was calculated using log-linear regression models. Absolute change represents the difference in mortality rate between 1990 and 2023.

Table S9. GBD 2023 Level 3 communicable diseases and rationale for exclusion from the present acute life-threatening infectious framework.

**Category**	**GBD Level 3 Cause**	**Rationale for Exclusion**
Chronic or long-latency systemic infections	Tuberculosis; HIV/AIDS; Chagas disease; Leishmaniasis; Schistosomiasis; Cysticercosis; Cystic echinococcosis; African trypanosomiasis	Characterized by chronic or prolonged clinical courses with distinct epidemiologic trajectories and care pathways; mortality typically reflects long-term disease progression rather than acute syndromic deterioration requiring emergency stabilization.
Vaccine-preventable or immunization-controlled infections	Measles; Varicella and herpes zoster; Diphtheria; Pertussis; Tetanus; Yellow fever; Rabies	Primarily addressed through immunization programs; although severe outcomes may occur, these conditions do not consistently represent dominant acute critical care syndromes among older adolescents at the global level.
Regionally concentrated or outbreak-driven infections	Ebola; Zika virus; Dengue; Invasive non-typhoidal Salmonella	Exhibit heterogeneous geographic distribution and outbreak-driven epidemiology; limited contribution to sustained global adolescent mortality patterns across the study period.
Generally mild or non–life-threatening infectious syndromes	Upper respiratory infections; Otitis media; Other intestinal infectious diseases; Intestinal nematode infections; Sexually transmitted infections excluding HIV; Acute hepatitis; Other unspecified infectious diseases; Other neglected tropical diseases	Typically associated with lower case-fatality risk or non–acute life-threatening trajectories in adolescents at the population level; not primarily characterized by rapid physiologic deterioration requiring emergency or critical care.

Note:

This table lists all Global Burden of Disease (GBD) 2023 Level 3 communicable diseases not included in the primary

analysis and summarizes the rationale for their exclusion. Causes were categorized according to differences in clinical trajectory, epidemiologic pattern, and relevance to acute, rapidly progressive syndromes typically requiring emergency or critical care in adolescents. Excluded conditions include chronic or long-latency infections, predominantly vaccine-controlled diseases, regionally concentrated or outbreak-driven infections, and infectious syndromes generally not characterized by rapid physiologic deterioration at the population level.
